# A Quantitative Sequencing Method for 5-Formylcytosine in RNA

**DOI:** 10.1002/ijch.202300111

**Published:** 2023-10-16

**Authors:** Ruitu Lyu, Kinga Pajdzik, Hui-Lung Sun, Linda Zhang, Li-Sheng Zhang, Tong Wu, Lei Yang, Tao Pan, Chuan He, Qing Dai

**Affiliations:** 1 Department of Chemistry, The University of Chicago, Chicago, IL 60637, USA.; 2 Department of Biochemistry and Molecular Biology, The University of Chicago, Chicago, IL 60637, USA.; 3 Howard Hughes Medical Institute, The University of Chicago, Chicago, IL 60637, USA.; 4 First Maternity & Infant Hospital, School of Medicine, Tongji University, Shanghai, China.

**Keywords:** 5-Formylcytosine, quantitative sequencing, transcriptome-wide, mutation rate, read-through rate

## Abstract

5-Formylcytosine (f^5^C) modification is present in human mitochondrial methionine tRNA (mt-tRNA^Met^) and cytosolic leucine tRNA (ct-tRNA^Leu^), with their formation mediated by NSUN3 and ALKBH1. f^5^C has also been detected in mRNA of yeast and human cells, but its transcriptome-wide distribution in mammals has not been studied. Here we report f^5^C-seq, a quantitative sequencing method to map f^5^C transcriptome-wide in HeLa and mouse embryonic stem cells (mESCs). We show that f^5^C in RNA can be reduced to dihydrouracil (DHU) by pic-borane, and DHU can be exclusively read as T during reverse transcription (RT) reaction, allowing the detection and quantification of f^5^C sites by a unique C-to-T mutation signature. We validated f^5^C-seq by identifying and quantifying the two known f^5^C sites in tRNA, in which the f^5^C modification fractions dropped significantly in ALKBH1-depleted cells. By applying f^5^C-seq to chromatin-associated RNA (caRNA), we identified several highly modified f^5^C sites in HeLa and mouse embryonic stem cells (mESC).

Over 100 naturally occurring RNA modifications have been identified so far, with some of them playing various roles in gene expression regulation^1–3^. As the most abundant internal modification in eukaryotic mRNA, *N*^6^-methyladenosine (m^6^A) is dynamically regulated and involved in numerous aspects of mRNA metabolism, such as alternative splicing^4^, nuclear export^5^, stability^6^, translation^7,8^ and decay^9^. In recent years, studies on transcriptome-wide sequencing of other mRNA modifications have also been emerging. The reported sequencing methods can be grouped as: (1) Antibody-based MeRIP-seq for m^6^A^4^, m^1^A^10–13^, ac^4^C^14,15^, m^5^C^16^ and hm^5^C^17^. These methods rely on antibody-based enrichment but could neither achieve base precision nor reveal absolute modification fraction. (2) Reverse transcription (RT) stop-based methods such as CMC-based pseudouridine sequencing^18^ and low dNTP-based 2′-*O*-Me sequencing^19^. While these methods can detect modification sites at base resolution, they usually have high false-positive rates since RT stop signatures could be generated non-specifically^20^. (3) RT mutation-based approaches, such as methods to map m^6^A^21–24^, m^7^G^25–27^ and m^1^A^28^ that generate mutation signatures at modified sites in order to achieve single base resolution with low background. (4) RT deletion-based approaches, such as BS-Induced quantitative pseudouridine sequencing^29,30^. Another consideration in RNA modification is the modification stoichiometry at each site. The modification fraction is a biological parameter that is directly related to the modification dynamics and their regulatory functions.

5-Methylcytosine (5mC), 5-hydroxylmethylcytosine (5hmC), and 5-formylcytosine (5fC) are DNA modifications that are important intermediates in an active DNA 5mC demethylation pathway. Sequencing methods for these modified bases in DNA have been well documented^31–37^. However, these modified bases also occur naturally in RNA, and their biological roles remain to be elucidated. m^5^C has been reported to protect RNA from degradation^38^, regulate mRNA export^39^, and promote the pathogenesis of bladder cancer^40^. Additionally, m^5^C on nuclear mRNA can serve as DNA damage codes to regulate DNA repair^41^. hm^5^C has been detected in mRNA^42^, and its presence was found to favor mRNA translation^17^. f^5^C displays approximately 100% modification fraction at C34 in mt-tRNA^Met43^ and a moderate modification fraction in ct-tRNA^Leu^ in human cells^43–45^. In both cases, NSUN3 was reported to be the methyltransferase that converts the target C to m^5^C, while ALKBH1 further catalyzes the oxidation of m^5^C to f^5^C^43^. f^5^C in tRNA is associated with several human diseases^46^ and f^5^C in tRNA-Leu-CAA promotes decoding under stress conditions^47^. In addition, the presence of f^5^C in yeast and human mRNA has also been detected by LC-MS/MS^48,49^. Here, we describe f^5^C-seq, a new method for quantitative sequencing of f^5^C in HeLa and mouse embryonic stem cells (mESCs). To detect 5fC in DNA, Zhu et al used malononitrile to specifically react with 5fC in DNA to generate a cyclized base which induces a C-to-T transition during DNA amplification^50^. Recently, a new method that employs pic-borane to reduce 5fC in DNA to DHU which could be read as T during amplification was reported^51^. We speculated that pic-borane reduction may also convert f^5^C base in RNA to DHU under optimized conditions, and RT enzyme may read through DHU efficiently and generate high C-to-T mutation rate to enable f^5^C detection and quantitation at base resolution in RNA.

To ascertain whether pic-borane facilitates the efficient and quantitative conversion of f^5^C to DHU in RNA, we initiated our study with the treatment of a 5-mer RNA oligo incorporating an f^5^C modification with pic-borane under different conditions (Table S1). The reactions were closely monitored utilizing MALDI-TOF MS method. We found that the reduction products were temperature dependent. At 25 °C, f^5^C was primarily reduced to dihydro-f^5^C (DHf^5^C) via 3,4-reduction, where DHU was obtained as the sole product at 70 °C via further deformylation and subsequent deamination (Figure 1a–b). Moreover, we detected a small peak at 1,529 Daltons at 25 °C, which represents the intermediate of 3,4-reduction and deformylation, but without deamination (Figure 1a–b). This observation suggests that deformylation occurs after 3,4-reduction and prior to deamination, which differs from the proposed mechanism for the pic-borane reduction of 5fC in DNA, in which deamination was thought to occur before deformylation^51^.

To determine whether RT enzymes can read through DHU and produce a C-to-T mutation in RNA, we undertook a primer extension on an f^5^C-containing 33-mer RNA oligo (Table S1), previously converted to its DHU counterpart (Figure 1c). Notably, both the untreated f^5^C-rich probe and the pico-borane treated sample rendered full-length products using the SuperScript II RT enzyme. In contrast, the sample treated with malononitrile predominantly produced RT-stop byproducts. The resulting cDNA products were then amplified by RT-PCR followed by Sanger sequencing. Our analysis revealed that untreated f^5^C was interpreted as C, while malononitrile treatment led to approximately 50% C-to-T mutations. Impressively, pico-borane treatment produced a significantly elevated C-to-T mutation rate of over 80% (Figure 1d). Collectively, our findings suggest that pico-borane-mediated conversion of f^5^C to DHU offers superior read-through and C-to-T mutation rates compared to malononitrile treatment. Additionally, we observed no significant RNA degradation when a 45-mer f^5^C-containing RNA oligo (Table S1) was treated with pico-borane across a temperature spectrum ranging from 55 to 70 °C (Figure S1). The mild nature of pico-borane treatment paved the way for the development of f^5^C-seq, which performs reduction after integrating the RNA fragments into library construction, followed by high-throughput sequencing (Figure S2).

We next investigated whether the C-to-T mutation rate is dependent on f^5^C sequence context and whether there is a significant linear correlation between the mutation rate and f^5^C fractions^11,25,28^. To do this, we treated fragmented small RNA isolated from HeLa cells treated with *E. coli* AlkB demethylase to remove the major tRNA methylations that block RT^52^. We then added spike-in oligos with NNf^5^CNN motifs (N represents a mixture of A, C, G and U) and five pairs of RNA oligos with different f^5^C modification fractions (Table S1). After performed 3′- and 5′-ligations, we treated the ligated RNA with pic-borane followed by RT reaction, PCR amplification, and sequencing to determine the C-to-T mutation rates of the reduced f^5^C. Our results showed that the C-to-T mutation rates were consistently high in all 256 NNf^5^CNN oligos, suggesting that the C-to-T mutation rate is generally independent of the f^5^C sequence context (Figure 2a). To our delight, we observed a nearly linear calibration curve, which allows us to precisely deduce the f^5^C modification fraction from the observed C-to-T mutation rate in RNA (Figure 2b).

In order to construct libraries suitable for sequencing, an alkaline fragmentation step is necessary. Initially, we performed MALDI TOF MS analysis of f^5^C-containing oligo treated in 0.1M NaHCO_3_ pH 9.2 at 95 °C for 9 min to evaluate the potential impact of alkaline fragmentation on f^5^C in RNA. Our data shows that f^5^C remains unaffected under alkaline fragmentation condition (Figure S3). Previous studies have shown that ALKBH1 catalyzes f^5^C formation in both mt-tRNA^Met^ and ct-tRNA^Leu^ in human cells^43^. Therefore, we used small RNA isolated from shControl and shALKBH1 HeLa cells to construct f^5^C-seq libraries (Figure S4a–b). We then examined the C-to-T mutation rates at the known f^5^C sites in tRNAs. In HeLa cells, we observed a high C-to-T mutation rate of approximately 80% at the mt-tRNA^Met^ f^5^C site and a low C-to-T mutation rate of around 15% at the ct-tRNA^Leu^ f^5^C site (Figure 2c, S5a), corresponding to f^5^C modification fractions of 87.2% and 16.4%, respectively, which is consistent with the previous reports based on mass spectrometry analysis^43^. The bases surrounding the f^5^C sites had minimal background mutation (Figure 2c). Two known f^5^C sites at tRNAs also showed very low C-to-U mutation rates in the input libraries (Figure S5). Additionally, we observed a marked reduction of f^5^C modification fractions in ALKBH1-deficient HeLa cells, while the mutation frequencies at adjacent cytosine sites remained unchanged upon ALKBH1 knockdown (Figure 2c, S5a). Similarly, our findings also revealed that both C34 sites in mt-tRNA^Met^ and ct-tRNA^Leu^ in mESCs are f^5^C-modified (Figure 2d, S5b), with a similar f^5^C modification fraction to that in the corresponding HeLa tRNAs. Notably, the f^5^C fractions decreased to nearly undetectable levels in ALKBH1-deficient mES cells (Figure 2d, S5b). Taken together, these findings robustly confirm the accuracy and quantitative reliability of our f^5^C-seq method in detecting f^5^C modifications at base resolution in RNA.

Given that f^5^C has previously been identified within human mRNA, we then tried to map transcriptome-wide f^5^C sites in polyA^+^ RNA isolated from both HeLa and mES cells using f^5^C-seq. Although we identified several hundred f^5^C sites in both cell lines, the f^5^C fraction at each site did not exceed 10%. Interestingly, when we employed f^5^C-seq on chromatin-associated RNA (caRNA) from HeLa and mES cells, we identified multiple f^5^C sites with high fraction levels (Table S2). This includes a site on the MER68 ERVL endogenous retrovirus-related Long Terminal Repeats (LTR) in HeLa cells (Figure 3a), and another on the U3 snRNA repeats in mES cells (Figure 3b). While the modification fraction at the MER68 ERVL LTR site remained relatively stable following ALKBH1 knockdown (Figure 3c), we observed a notable reduction at the U3 snRNA repeat site. Specifically, upon ALKBH1 depletion, the modification fraction declined markedly from 39.04% to 26.62%, which corresponds to a decrement in the f^5^C fraction from 42.57% to 29.27% (Figure 3d). This data presents a compelling avenue for further exploration into the dynamic roles and regulation of f^5^C modifications in RNA biology.

It is worth to mention that during the preparation of this manuscript, several other RNA f^5^C sequencing methods have been published^53–55^. One of these methods uses pyridine borane as a reductant^53^, while the other was based on the selective and efficient malononitrile-mediated labeling of f^5^C residues to generate adducts that are read as C-to-T mutations upon reverse transcription^54^. However, our f^5^C-seq method distinguishes itself by utilizing pic-borane as a reductant, akin to the pyridine borane used in published method. Through extensive analysis, we demonstrated that pic-borane can proficiently reduce f^5^C to DHU, similarly inducing C-to-T transitions at f^5^C sites during RT-PCR, which facilitates f^5^C single-base resolution detection. When contrasted with other methods that employ malononitrile or photo-mediated labeling, our technique stands out for its simplicity and efficiency in mapping transcriptome-wide f^5^C sites. These newly developed methods represent exciting developments in the field and offer alternative approaches to sequencing f^5^C modifications. The emergence of multiple methods for detecting f^5^C modifications highlights the growing interest in this area of research and suggests that there is still much to be learned about the function and regulation of these modifications in various cellular contexts. As the field continues to evolve, it will be important to compare the strengths and limitations of different approaches and to identify the best methods for studying f^5^C modifications in different biological systems.

In summary, we have developed f^5^C-seq, a quantitative sequencing method for mapping f^5^C modification in RNA. Our method is based on the chemical principle that f^5^C in RNA can be specifically and quantitatively reduced to DHU by pic-borane at a higher temperature, and DHU is read as T instead of C in RNA sequencing. It is worth to note that although in principle ca^5^C can also be converted to DHU by pic-borane to generate C to T mutation, so far, no ca^5^C has been detected in RNA. Using f^5^C-seq, we verified the two known f^5^C sites of mt-tRNA^Met^ (C34) and ct-tRNA^Leu^ (C34) in human tRNA and confirmed that their f^5^C modification fractions are sensitive to ALKBH1 knockdown. Further sequencing confirmed that ALKBH1 is also responsible for the formation of these two f^5^C sites in mES cells. We then sequenced f^5^C in HeLa and mESCs polyA+ RNA. The f^5^C levels in identified hundreds of sites were low and did not exceed 10%. This result is consistent with the low f^5^C levels (~1.7 ppm) measured in HEK293C polyA+ RNA by LC-MS/MS by Arguello et al^56^. We also applied f^5^C-seq to caRNA from HeLa and mES cells and detected several highly modified f^5^C sites that were not reported previously. We found that f^5^C located on mouse U3 snRNA repeats was sensitive to ALKBH1 depletion, suggesting that ALKBH1 is also responsible for f^5^C formation at this position. Interestingly, the f^5^C fraction identified on human MER68 ERVL LTR did not change upon ALKBH1 KD. Further studies are needed to unravel the enzyme responsible for the formation of f^5^C on human MER68 ERVL LTR. Other RNA modifications, notably m^6^A, have been co-transcriptionally integrated into various caRNAs in mammalian cells. These modifications play a pivotal role in controlling RNA abundance, which in turn influences gene transcription through alterations in chromatin accessibility^57^. Intriguingly, in our studies, we have identified multiple sites on caRNA with pronounced f^5^C modifications in both HeLa and mouse ES cells. Noteworthy among these are the f^5^C sites present on the carRNA MER68 ERVL LTR and U3 snRNA repeats. These f^5^C sites exhibit diverse responses to ALKBH1 KD, which hints at the potential diverse roles of f^5^C in orchestrating chromatin states, influencing transcription, and governing alternative splicing. This diverges from its established regulatory function in translation. Furthermore, given the prevalence of m^5^C sites on both caRNA^58^ and mRNA^59^, we speculate that f^5^C could serve as an intermediate in a potential RNA demethylation process. Taken together, f^5^C-seq provides a quantitative tool for future studies on the biological function of f^5^C in RNA.

## Supplementary Material

Supp Information

## Figures and Tables

**Figure 1.**
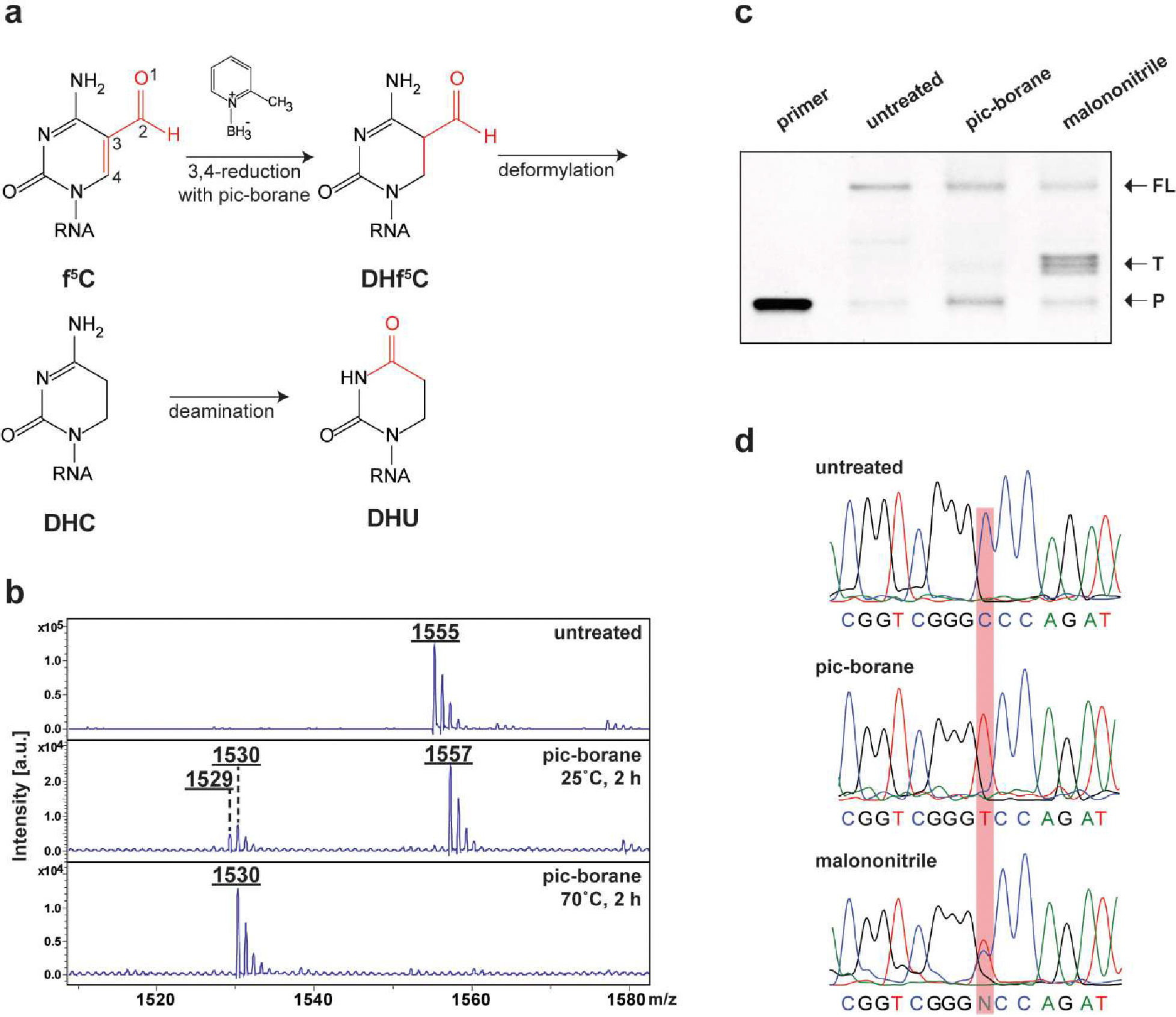
f^5^C-seq and its chemical validation. a Suggested pic-borane f^5^C reduction mechanism based on the observed intermediates. b MALDI-TOF MS analysis comparing an untreated f^5^C-continaing RNA probe with the same probe treated with pic-borane for 2 h at either 25 °C or 70 °C. The observed peaks at m/z values 1,555, 1,557, 1529 and 1,530 represent oligos integrated with f^5^C, DHf^5^C, DHC and DHU, respectively. Notably, the peak at 1,557 represents the intermediate that f^5^C is reduced via 3,4-reduction and undergoes subsequent deformylation process, yielding DHC followed by further deamination to produce DHU. c Primer extension assay of 33-mer f^5^C-containing RNA oligo treated with pic-borane and malononitrile. FL: full length; T: truncated product; P: primer. d Sanger sequencing of untreated, pic-borane and malononitrile treated f^5^C-containing 33-mer RNA oligos followed by RT-PCR.

**Figure 2.**
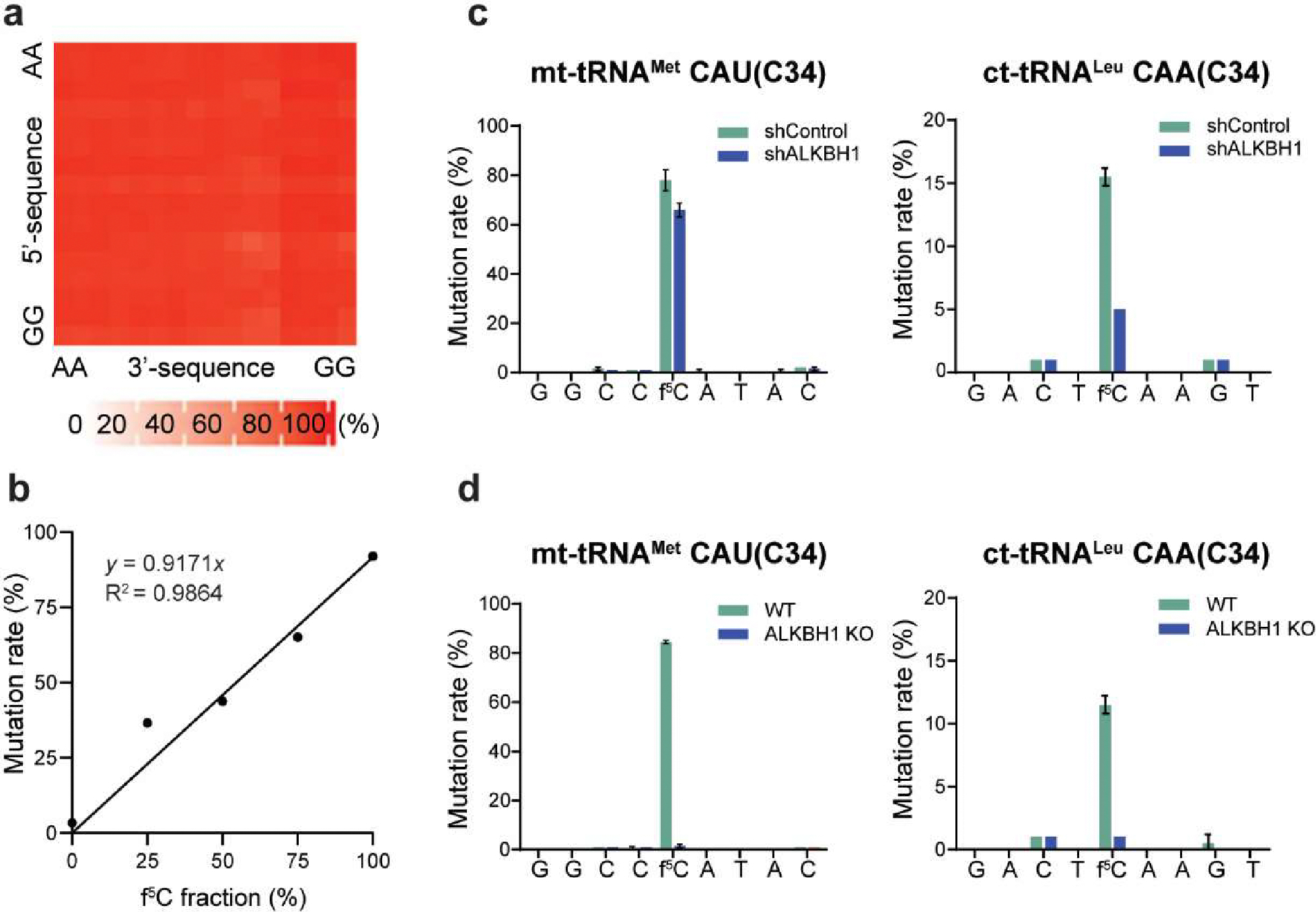
Validation of f^5^C-seq by identifying two known f^5^C sites in human tRNA with next generation sequencing. a Mutation rate is independent of sequence context around the f^5^C site. b Calibration curve of spike-in oligos containing f^5^C with varying f^5^C fractions and C-to-T mutation rates. c C-to-T mutation rates of mt-tRNA^Met^ CAU(C34) and ct-tRNA^Leu^ CAA(C34) sites, as well as their neighboring sites, in shControl and shALKBH1 HeLa cells. d C-to-T mutation rates of mt-tRNA^Met^ CAU(C34) and ct-tRNA^Leu^ CAA(C34) sites, as well as their neighboring sites, in WT and ALKBH1-KO mESC. Bars represent mean of two technical replicates ± SD. Statistical significance was determined by t-test using the Holm-Sidak method.

**Figure 3.**
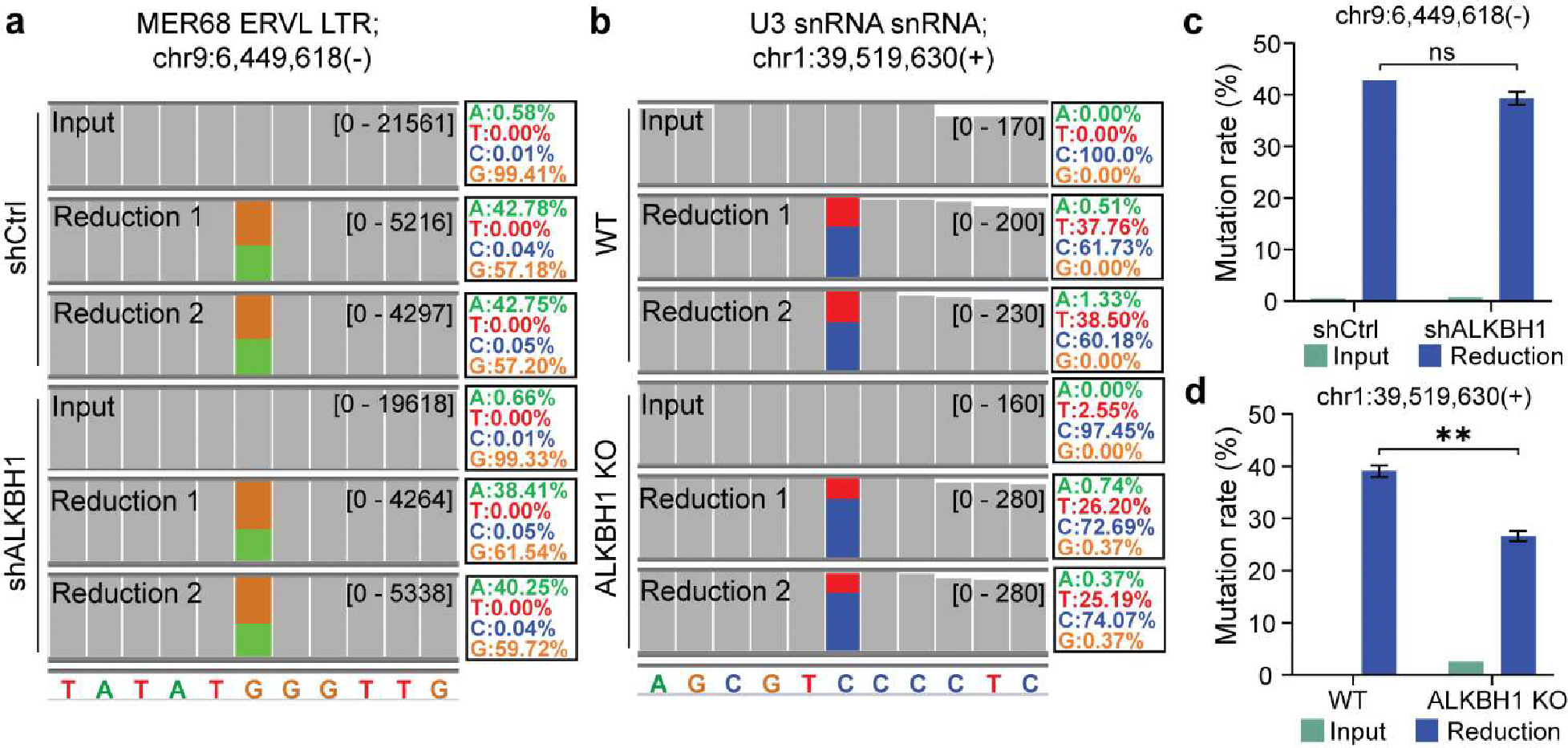
Overview of f^5^C sites detected in caRNA in HeLa and mES cells. a IGV tracks showing the mutation signature of the identified f^5^C site on caRNA MER68 ERVL LTR in shCtrl and shALKBH1 HeLa cells. b IGV tracks showing the mutation signature of identified f^5^C site on caRNA U3 snRNA repeats in wide-type (WT) and ALKBH1 knockout (KO) mES cells. c C-to-T mutation rates of detected tRNA f^5^C site on caRNA MER68 ERVL LTR in shCtrl and shALKBH1 HeLa cells. Statistical significance was determined by t-test using the Holm-Sidak method (ns: not significant). d C-to-T mutation rate of detected f^5^C site on caRNA U3 snRNA repeats in WT and ALKBH1 KO mES cells. Bars represent mean of two technical replicates ± SD. Statistical significance was determined by t-test using the Holm-Sidak method (**p < 0.01).
