# Supplementary material for: A Quantitative Sequencing Method for 5-Formylcytosine in RNA
Source: Isr J Chem. Author manuscript; Available in PMC 2025 Jun 9. (PMC12147524; doi:10.1002/ijch.202300111)
Supplement: Supp Information [file NIHMS2063182-supplement-Supp_Information.pdf]

Supporting Information

A Quantitative Sequencing Method for 5-Formylcytosine in RNA

Ruitu Lyu[1,‡], Kinga Pajdzik[1,2,‡], Hui-Lung Sun[1], Linda Zhang[1], Li-Sheng Zhang[1], Tong Wu[1], Lei Yang[4], Tao Pan[2,*], Chuan He[1,2,3,*], Qing Dai[1*]

[1]Department of Chemistry, [2]Department of Biochemistry and Molecular Biology, [3]Howard Hughes Medical Institute, The University of Chicago, Chicago, IL 60637, USA. [4]First Maternity & Infant Hospital, School of Medicine, Tongji University, Shanghai, China.

‡These authors contributed equally

Contents

Figure S1. f^5C-containing oligo was stable with pic-borane treatment. 2

Figure S2. Schematic outline of library construction for f^5C-seq. 3

Figure S3. Alkaline fragmentation conditions do not affect f^5C 4

Figure S4. Western blots of ALKBH1 expression. 5

Figure S5. IGV of two f^5C sites in human and mouse tRNA 6

Table S1. DNA and RNA oligonucleotides 7

Table S2. Detected f^5C sites in caRNA in HeLa and mESC 7

Methods 8

Supporting references 11

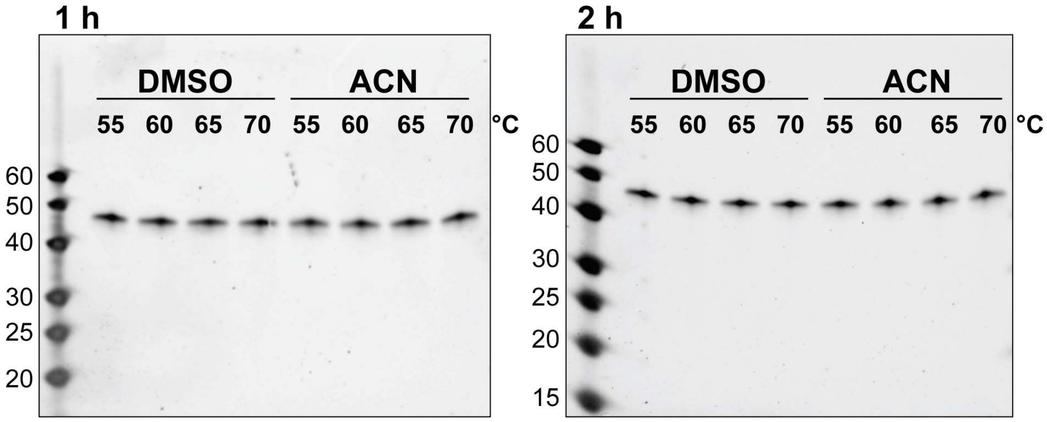

Figure S1. f⁵C-containing oligo was stable with pic-borane treatment. The gels of 45-mer f^5C-containing oligo treated with pic-borane diluted in DMSO or acetonitrile (ACN). RNA oligo was treated for 1 or 2 hours at different temperatures ranging from 55 to 70 °C. ssDNA was used as a ladder.

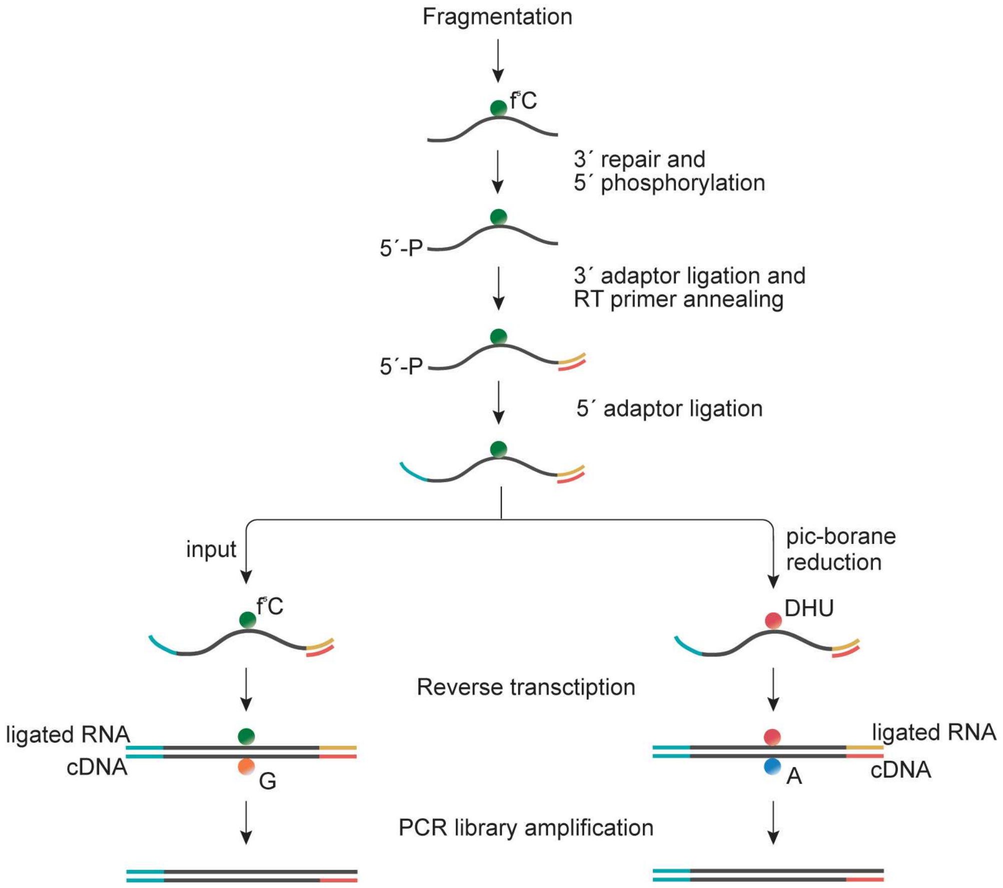

Figure S2. Schematic outline of library construction for f⁵C-seq.

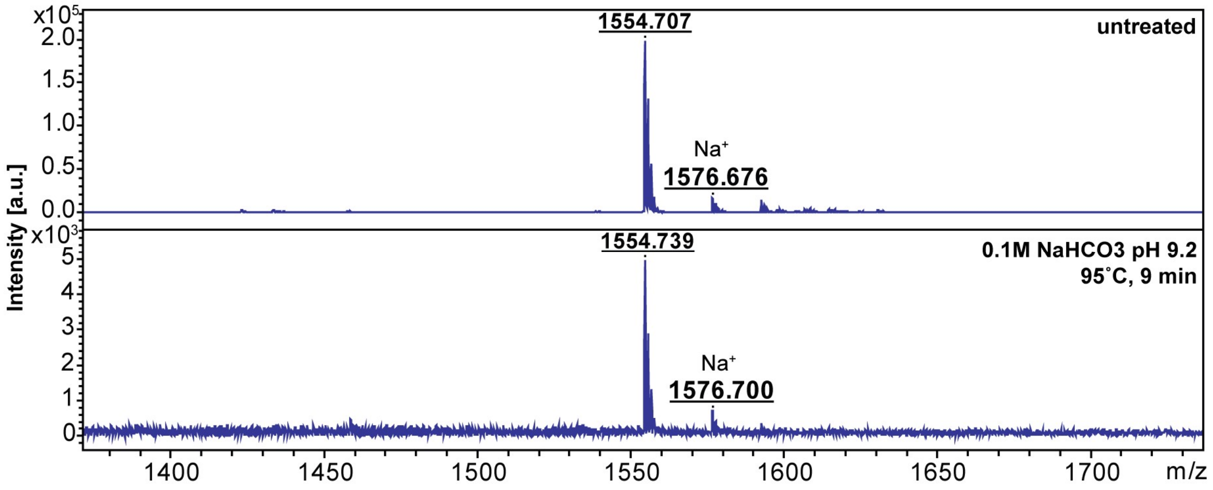

Figure S3. Alkaline fragmentation conditions do not affect f^5C
MALDI TOF MS analysis for f^5C-containing oligo treated in 0.1M $NaHCO_3$ pH 9.2 at 95 °C for 9 min.

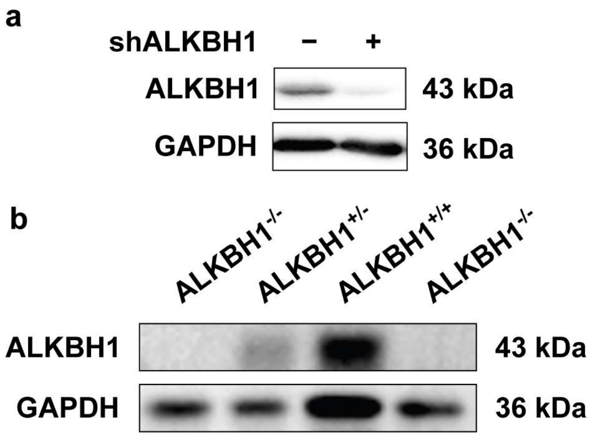

Figure S4. Western blots of ALKBH1 expression.
(a) Western blots showing ALKBH1 expression in shControl and shALKBH1 in HeLa cells. (b) Western blots showing ALKBH1 expression in WT and ALKBH1 KO in mESC cells.

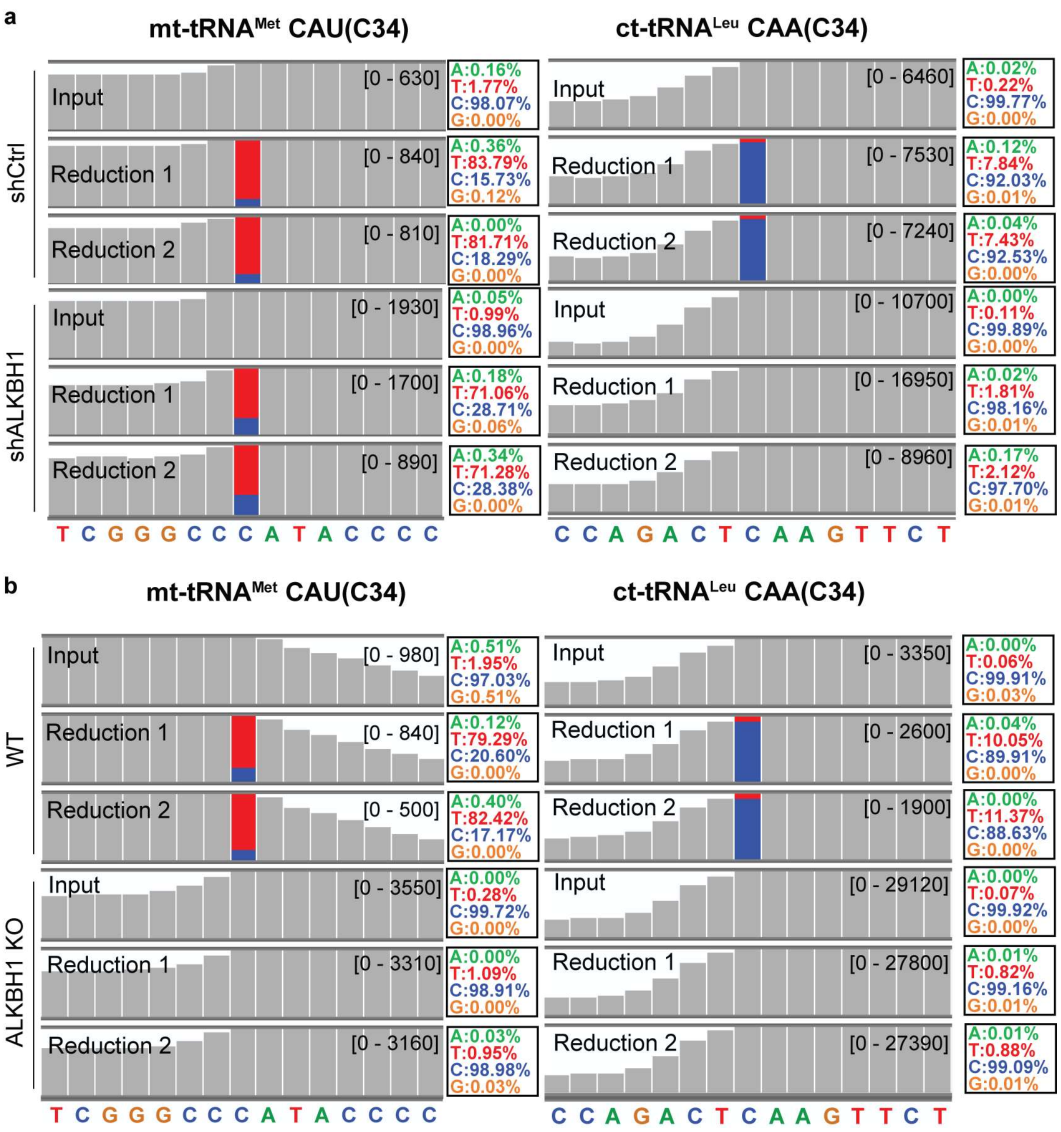

Figure S5. IGV of two f^{5}C sites in human and mouse tRNA
(a) IGV coverage traces of mutation signatures of two known f^{5}C sites on human mt-tRNA^{Met} CAU(C34) and ct-tRNA^{Leu} CAA(C34) in HeLa cells. (b) IGV coverage of mutation signatures of two known f^{5}C sites on mouse mt-tRNA^{Met} CAU(C34) and ct-tRNA^{Leu} CAA(C34) in mESC cells.

Table S1. DNA and RNA oligonucleotides

Oligo name	Sequence 5′→3′
**5-mer**	ACf^{5}CGU
**33-mer**	GGAGACGGUCGGGf^{5}CCCAGAUAUUCGUAUCUGUC

**45-mer**	CCUCACCAUCUCAACCUGAGUCGf^{5}CGGCAUGAGGGAGAAGUGGUGA
**FAM primer**	FAM/CGGAGCCCACACTCTACTCGACAGATACGAATAT
**RT primer**	GCGGAGCCCACACTCTACTCGACAGATACGAATAT
**PCR primer-F**	CTAATACGACTCACTATAGGGCGGAGACGGTCGGG
**PCR primer-R**	TTTGCTGAGGAGTGCCGTTAATTAAGTGCGGAGCCCACACTCT
**Sanger sequencing primer**	TGCTGAGGAGTGCCGT
**25% f^{5}C**	UGUCAUCACGACCf^{5}CAUAUAAGCUGACU
**50% f^{5}C**	UGUCCGAUGUACCf^{5}CAUAUAAGCUGACU
**75% f^{5}C**	UGUCUUAGGCACCf^{5}CAUAUAAGCUGACU
**100% f^{5}C**	UGUCUGACCAACCf^{5}CAUAUAAGCUGACU
**25% C**	UGUCUUAGGCACCCAUAUAAGCUGACU
**50% C**	UGUCCGAUGUACCCAUAUAAGCUGACU
**75% C**	UGUCAUCACGACCCAUAUAAGCUGACU
**100% C**	UGUCGCCAAUACCCAUAUAAGCUGACU
**NN-f^{5}C-NN**	CAU ACU CNN f^{5}CNN GGA CUA CAG CUG CUC GAU
**3′ adaptor**	5’rApp-NNNNNATCACGAGATCGGAAGAGCACACGTCT/3BioTEG
**5′ adaptor**	GUUCAGAGUUCUACAGUCCGACGAUCNNNNNU

Table S2. Detected f^{5}C sites in caRNA in HeLa and mESC

	Location	Mutation rate (%)	Estimated fraction (%)
HeLa	chr9:6449618-6449619 (-)	42.81	46.68
	chr21:9827090-9827091 (+)	10.78	11.75
	chrX:118557777-118557778 (+)	10.60	11.56
mESC	chr1:39519630-39519631 (+)	39.04	42.57
	chr6:49236524-49236525 (+)	10.54	11.49
	chr6:3201474-3201475 (+)	9.82	10.71

DNA and RNA oligonucleotides

PCR primers and the primer labeled with 5′-FAM were purchased from Integrated DNA Technologies, Inc. (IDT). Other oligonucleotides were synthesized in house.

Methods

Monitoring pyridine borane reduction of f^{5}C in RNA by MALFI-TOF MS

5-mer RNA oligonucleotide containing f^5C was diluted in 40 µL water. Then, 5 µL of 3 M NaOAc pH 5.2 was added and the mixture was incubated under different temperature for 5 min. Next, without removing the samples from the PCR machine, 5 µL of 5 M 2-methylpyridine borane complex (Sigma-Aldrich, #654213) diluted in acetonitrile was added. After incubation for 2 h, 1 µL of reaction mixture was taken out to mix with matrix and loaded onto plate. Negative reflector mode was used to measure MS.

Primer extension assay

100 ng of 33-mer RNA oligonucleotide containing f^5C was treated with pic-borane or malononitrile (Alfa Aesar, #A15046). For pic-borane treatment, RNA was diluted in 40 µL water. Then, 5 µL of 3 M NaOAc pH 5.2 was added and the mixture was incubated at 65 °C for 5 min. Next, without removing the sample from the PCR machine, 5 µL 5 M pic-borane diluted in acetonitrile was added and incubated for 65 °C 1 h. As for malononitrile treatment, RNA was treated with 150 mM malononitrile in 100 mM Tris pH 7.5 at 37 °C for 16 h. After the treatment, RNA was purified by Oligo Clean & Concentrator (Zymo Research), and 40 ng purified RNA was used for RT reaction. 1 µL of 1 µM FAM-primer was added to RNA and annealed at 65 °C for 5 min, then put on ice for 2 min. Then, 2 µL of 5x SSII buffer, 0.5 µL of 10 mM dNTP, 1 µL of 0.1 M DTT, 0.25 µL of Murine RNase inhibitor (NEB, #M0314), 0.5 µL of SSII (Invitrogen™, #18064022) and RNase-free water to 10 µL were added and incubated at 42 °C for 1 h followed by 80 °C for 10 min. After RT reaction, 2.5 µL of the reaction mixture was incubated with 2.5 µL 2x RNA loading dye and incubated at 95 °C for 5 min and immediately load onto 15% denaturing polyacrylamide gel and run at 180 V for 90 min. Then, the gel was imaged by Bio-Rad ChemiDoc Imager.

Mutation rate assay

100 ng of 33-mer RNA oligonucleotide containing f^5C was treated with pic-borane or malononitrile. For pic-borane treatment, RNA was diluted in 40 µL water. Then, 5 µL of 3 M NaOAc pH 5.2 was added and the mixture was incubated at 65 °C for 5 min. Next, without removing the sample from the PCR machine, 5 µL of 5 M pic-borane diluted in acetonitrile was added and incubated for 65 °C 1 h. As for malononitrile treatment, RNA was treated with 150 mM malononitrile in 100 mM Tris pH 7.5 at 37 °C for 16 h. After the treatment, RNA was purified by Oligo Clean & Concentrator (Zymo Research), and 10 ng purified RNA was used for RT reaction. RNA was annealed with 1 µL of 10 µM RT primer at 65 °C for 5 min and then put on ice for 2 min. Then, 4 µL of 5x SSII buffer, 1 µL of 10 mM dNTP, 2 µL of 0.1 M DTT, 1 µL of murine RNase inhibitor (NEB, #M0314), 1 µL of SSII (Invitrogen™, #18064022) and RNase-free water to 20 µL were added and incubated at 42 °C for 1 h followed by 80 °C for 10 min. Then, 2 µL cDNA was used to carry out PCR reaction with Taq DNA Polymerase (NEB, #M0320), according to vendor's instructions. To analyze the C to U mutation, PCR product was then sequenced by Sanger sequencing.

Degradation assay

80 ng of 45-mer RNA oligonucleotide containing f^5C was diluted in 40 µL water. Then, 5 µL of 3 M NaOAc pH 5.2 was added and the mixture was incubated under different temperature for 5 min. Next, without removing the samples from the PCR machine, 5 µL of 5 M pic-borane diluted in acetonitrile or DMSO was added. After incubation for 1 or 2 h, RNA was purified by Oligo Clean & Concentrator (Zymo Research), mixed with 2x RNA loading dye (9M urea, 100 mM EDTA, 0.2% (w/v) bromophenol blue) and loaded onto 15% denaturing polyacrylamide gel (Invitrogen™, #EC6885BOX) and run at 180 V for 90 min. Then, the gel was stained with SYBR™ Gold Nucleic Acid Gel Stain (Invitrogen™, #S11494) and imaged by ChemiDoc Imager (Bio-Rad).

Mouse ES cell line derivation

Alkbh1+/- female mice (8-12 weeks) were super-ovulated and mated with Alkbh1+/- male mice. Zygotes were collected from oviducts and cultured in G1 plus medium for 3 days until the blastocyst stage. Blastocysts were collected and seeded onto the feeder in a 96-well plate and cultured in derivation medium: knockout DMEM, 20% knockout serum replacement, nucleosides (100x), 1 mM L-glutamine, 1% nonessential amino acid stock, 0.1 mM 2-mercapto-ethanol and 1,000 U/mL LIF for 7-10 days. The outgrowth from the blastocysts was then passaged in 24-well plates for regular ESC culture using ES medium: DMEM, 15% fetal bovine serum, nucleosides (100x), 1 mM l-glutamine, 1% nonessential amino acid stock, 0.1 mM 2-mercaptoethanol, 1,000 U/mL LIF, 3 mM CHIR99021 and 1 mM PD0325901.

Fragmentation of HeLa and mESC small RNA and AlkB treatment.

200-300 ng of cellular small RNA (size < 200 nt) was fragmented into 40-50 nt using in 0.1M $NaHCO_3$ pH 9.2 at 95 °C for 9 min and purified by Oligo Clean & Concentrator (Zymo Research). Then RNA was subjected to demethylation treatment with 1 µL of engineered AlkB (D135S, 10 mg/mL) with the reported recipe[1], followed by purification using Oligo Clean & Concentrator (Zymo Research).

Fragmentation of HeLa and mESC caRNA

200-300 ng of caRNA was fragmented into 40-50 nt using in 0.1M $NaHCO_3$ pH 9.2 at 95 °C for 9 min and purified by Oligo Clean & Concentrator (Zymo Research).

Library preparation for next generation-sequencing

1. **3′-repair and 5′-phosphorylation:** To fragmented RNA, RNA water was added to 38 µL, 5 µL of T4 PNK 10x buffer and 1 µL T4 PNK enzyme (NEB, #M0201)) were added and the mixture was incubated at 37 °C for 30 min. Then 5 µL of ATP (10 mM) and 1 µL of T4 PNK enzyme were added, and the mixture was further incubated at 37 °C for 1 h, followed by incubation at 65 °C for 20 min to inactivate the enzyme, followed

by purification using Oligo Clean & Concentrator (Zymo Research) eluting with 7 µl RNase-free water.

2. **3′-Ligation:** 100 ng of fragmented RNA was diluted in 6 µL. Then, 1 µL of 11.25 µM 3′-adaptor was added and the mixture was incubated at 70 °C for 2 min and then immediately put on ice. After 5 min, 10 µL of 3′-ligation buffer (2x) and 3 µL of 3′-ligation enzyme mix (NEBNext Multiplex Small RNA Library Prep Set for Illumina, NEB #7300) were added and the mixture was incubated at 16 C for 16 h.
3. **RT primer annealing:** To the above mixture, 4.5 µl RNA water and 1 µL of RT primer (NEBNext Multiplex Small RNA Library Prep Set for Illumina, NEB #7300), and the mixture was incubated at 75 °C for 5 min, 37 °C for 15 min and 25 °C for 15 min.
4. **5′-Ligation:** n x 1.1 µL of 11.25 µM 5′-adaptor was incubated at 70 °C for 2 min and then immediately put into ice for 5 min. Then, 1 µL of 5′-ligation buffer and 2.5 ul of 5′-ligation enzyme mixture (NEBNext Multiplex Small RNA Library Prep Set for Illumina, NEB #7300) were added. The mixture was incubated at 25 °C for 16 h and purified by Oligo Clean & Concentrator (Zymo Research), eluting with 16 µl water.
5. **Pic-borane treatment:** 2.5 µL ligated RNA was diluted in 40 µL water. Then, 5 µL of 3 M NaOAc pH 5.2 was added and the mixture was incubated at 65 °C for 5 min. Next, without removing the samples from the PCR machine, 5 µL of 5 M pic-borane diluted in acetonitrile was added and incubated at 65 °C for 1 h. Next, RNA was purified by Oligo Clean & Concentrator (Zymo Research) and eluted with 14 µL RNase-free water.
6. **RT reaction:** For treated libraries, to 12 µL of pyridine borane treated RNA was added 4 µL of 5x SSII buffer, 1 µL of 10 mM dNTP, 2 µL of 0.1 M DTT, 1 µL of murine RNase inhibitor (NEB, #M0314), 1 µL of SSII (Invitrogen™, #18064022) and RNase-free water to 20 µL and incubated at 42 °C for 1 h followed by 80 °C for 10 min. For input library, to 2 µL ligated RNA without pyridine borane treatment RNase-free water was added to 10 µL and followed the same procedure to conduct RT reaction.
7. **qPCR:** Took 1 µL RT product and added 7 µL water, 1 µL of 10 µM index primer and 1 µL of 10 µM SR primer (NEBNext Multiplex Small RNA Library Prep Set for Illumina, NEB #7300) and 10 µL FS Essential Green Master Mix (Roche, 6924204001). The mixture was subjected to qPCR to determine the PCR cycles.
8. **PCR:** To a 200 µL PCR-tube were added 12.5 µL LongAmp Taq 2x Master mix, 0.625 µL SR Primer for Illumina, 0.625 µL index primer (NEBNext Multiplex Small RNA Library Prep Set for Illumina, NEB #7300), 1.25 µL water and 10 µL of cDNA. The mixture was incubated in a 94 °C for 30 sec, followed by n cycles of 95 °C for 15 sec, 62 °C for 30 sec, 70 °C for 15 sec, and then 5 min at 70 °C.
9. **Purification of libraries by agarose gel:** Libraries were loaded onto low melting agarose gel and run at 100 V for 75 min in 1x TBE buffer, and the desired bands were cut under UV. The libraries were extracted from the gel using MinElute Gel Extraction Kit (Qiagen, #28604).

Next generation-sequencing data processing

1. Spike-In oligos sequencing data processing.

Low-quality and adapter-containing reads were trimmed from Spike-In oligos raw sequencing data using trim-galore package in single-end mode. Reads shorter than 50 bp were removed. Then, we used fastx_collapser in FASTX-toolkit to collapse identical sequences in a fastq file into a single sequence, which was used to remove duplicated reads from PCR amplification. For the reason of In-line barcode design in the Spike-In libraries, first 5bp bases and last 11bp bases were cut from the collapsed reads. The modified reads were finally used to calculate mutation rates based on different sequence context and generate the calibration curve.

2. Small RNA and PolyA$^+$ RNA sequencing data processing.

We used the same procedure and tools for small RNA and polyA$^+$ RNA sequencing data pre-processing. The modified reads were aligned to the reference genome (hg19 for HeLa cells or mm10 for mESC) using bowtie2 (v2.3.3.1)(small RNA) and tophat2(PolyA$^+$ RNA) under default parameters. Mapped sam files were subsequently converted and sorted to bam files using samtools sort (v1.9). Sorted bam files were subsequently filtered to get the unique mapped reads using samtools view (-q 5). Rnaseqmut was used to identify mutations under the "-t -s 2 -m 4" parameters. Two cutoffs were used to filter the mutation list identified by rnaseqmut for downstream analysis: 1) reads coverage equal to or greater than 50 and mutation ratio less than 4% in Input library, 2) reads coverage equal to or greater than 100 and mutation ratio equal to or greater than 4% in both pic-borane treated libraries.

Supporting references

(1) Zheng, G.; Qin, Y.; Clark, W. C.; Dai, Q.; Yi, C.; He, C.; Lambowitz, A. M.; Pan, T. Efficient and Quantitative High-Throughput TRNA Sequencing. *Nat. Methods* **2015**, *12* (9), 835–837. https://doi.org/10.1038/nmeth.3478.
